# Growing and cultivating the forest genomics database, TreeGenes

**DOI:** 10.1093/database/bay084

**Published:** 2018-09-18

**Authors:** Taylor Falk, Nic Herndon, Emily Grau, Sean Buehler, Peter Richter, Sumaira Zaman, Eliza M Baker, Risharde Ramnath, Stephen Ficklin, Margaret Staton, Frank A Feltus, Sook Jung, Doreen Main, Jill L Wegrzyn

**Affiliations:** 1 Department of Ecology and Evolutionary Biology, University of Connecticut, Storrs, CT, USA; 2 Department of Horticulture, Washington State University, Pullman, WA, USA; 3 Department of Entomology and Plant Pathology, University of Tennessee, Knoxville, TN, USA; 4 Department of Genetics and Biochemistry, Clemson University, Clemson, SC, USA

## Abstract

Forest trees are valued sources of pulp, timber and biofuels, and serve a role in carbon sequestration, biodiversity maintenance and watershed stability. Examining the relationships among genetic, phenotypic and environmental factors for these species provides insight on the areas of concern for breeders and researchers alike. The TreeGenes database is a web-based repository that is home to 1790 tree species and over 1500 registered users. The database provides a curated archive for high-throughput genomics, including reference genomes, transcriptomes, genetic maps and variant data. These resources are paired with extensive phenotypic information and environmental layers. TreeGenes recently migrated to Tripal, an integrated and open-source database schema and content management system. This migration enabled developments focused on data exchange, data transfer and improved analytical capacity, as well as providing TreeGenes the opportunity to communicate with the following partner databases: Hardwood Genomics Web, Genome Database for Rosaceae, and the Citrus Genome Database. Recent development in TreeGenes has focused on coordinating information for georeferenced accessions, including metadata acquisition and ontological frameworks, to improve integration across studies combining genetic, phenotypic and environmental data. This focus was paired with the development of tools to enable comparative genomics and data visualization. By combining advanced data importers, relevant metadata standards and integrated analytical frameworks, TreeGenes provides a platform for researchers to store, submit and analyze forest tree data.

## Introduction

The availability of high-throughput sequencing has enabled the assembly of over 4500 eukaryote genomes in the past 10 years (8). Despite this tremendous progress, fewer than 300 are land plant genomes and only 26 represent forest tree species (29). The vast majority of genomics data are generated in the form of transcriptomes, targeted sequencing such as exome capture, or genotyping-by-sequencing (GBS). In addition to limitations of reference material, traditional repositories for genomics data, including plant genomics, provide limited access, curation and integration for non-model organisms. Repositories focused on the plant kingdom, such as the Joint Genome Institute’s (JGI) Phytozome (9), serve as the gold standard for reference plant genomes but do not integrate beyond the genome sequence and the associated annotation. A related challenge surrounding high-throughput technologies is the cost required to store this information. The cost to keep a single base pair on local hardware or in the ‘cloud’ has now exceeded the expense associated with sequence generation (26). Primary repositories must decide what types of data are most critical for their user base and how to provide this in a value-added context. For example, NCBI’s Genbank is no longer accepting variant data that is not associated with biomedical models (8). For plants, and other organisms, it is the role of the clade and model organism databases to fill these voids for data collection and provide a platform by which these data can be integrated and queried in a manner that is consistent with the objectives of the research community.

**Figure 1:**
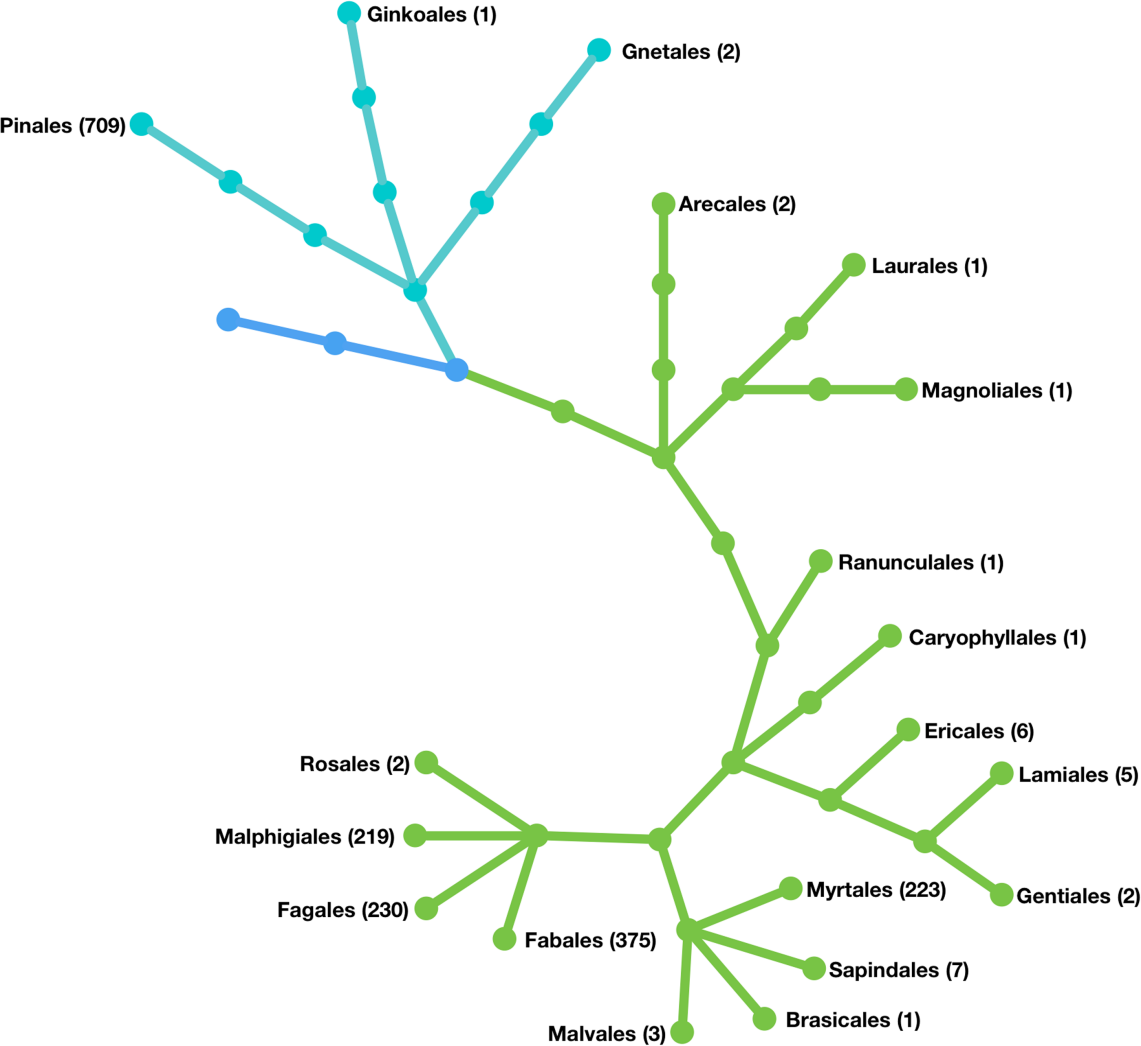
Phylogenetic representation of the forest tree orders held in the TreeGenes database. The orders are listed and the number of species in each are included parenthetically. Cyan branches represent gymnosperm orders; green branches represent angiosperm orders.

TreeGenes is a clade organism database for forest trees, representing 16 orders and over 1790 species (Figure 1). This repository stores and curates genetic information and the related phenotypic and environmental metrics to provide value to various end users. The data consumers of TreeGenes represent academic research labs, commercial breeders, government agencies and a variety of foundations. Although their objectives vary, they share a common interest in integrating data across scales to develop approaches for assisted migration (1), reforestation (24), marker-assisted breeding (21) and pathogen management (23) at the population level. Population scale studies are aimed at improving and understanding forest tree populations through comparative genomics, ecological genomics and quantitative genetics (25). While genomics technologies are enabling these endeavors, the resources available for specific species vary as widely as the organizations focused on them (22).

Since its inception in the 1990s as a framework to store genetic maps, the TreeGenes database has grown to hold nearly every genetic and genomic data type available (27, 28). Recent collaborations with large-scale research projects have mandated that phenotypic data be stored in a relational manner alongside these genomics datasets. Moreover, it has become apparent that environmental data are also needed to answer questions relevant to threatened forests and breeding populations. The diverse research interests of the community, numerous species studied and wide-ranging technologies require extensive metadata in order to integrate and analyze the heterogeneous data downstream. Beyond data integration, the necessity to store novel data types and implement new standards as the field rapidly changes requires a flexible, modular and standardized framework. Towards this end, TreeGenes has become a part of the federation of Tripal databases and has developed modules focused on cross-site querying, data transfer and direct access to analytical tools that serve the unique forest tree community.

### Software architecture of TreeGenes

#### TreeGenes’ underlying Tripal framework

TreeGenes is an early adopter of the Tripal v3 framework to organize and integrate data, develop new applications and coordinate data exchange. This open source toolkit facilitates the construction of high quality, standards-based, genetic databases. The Tripal platform, first released in 2008, currently supports over 120 sites for a wide range of species (2). Tripal unifies the Generic Model Organism Database schema known as Chado (3) with the content management system, Drupal (https://www.drupal.org/). The core Tripal installation provides a series of templates to store and query common sequence types such as genes, proteins and genome sequences, as well as publications. This union of web and database tools provides a framework that can be easily deployed and expanded. Tripal encourages database managers to develop and share extension modules that can provide additional features for new data types or more efficient data access. Recent development of Tripal v3 involved a collaboration among TreeGenes, Hardwood Genomics Project (6), Genome Database for *Rosaceae* (4), Citrus Genome Database (https://www.citrusgenomedb.org) and the Legume Federation (https://legumefederation.org) to enable cross-database communication and connection to analytical pipelines through three core modules, Tripal Exchange, Tripal Galaxy and Tripal Transfer, branded as Tripal Gateway.

In cases where the database schema diverges from standard nomenclature or holds new data types, Tripal Ex-change (http://tripal.info/tutorials/v3.x/web-services) provides greater flexibility for local architectures through web services. This feature permits each Tripal database to define their terms, and therefore provides a mechanism to search the same data types across Tripal databases, regardless of how they are stored. An extension on this framework developed by Hardwood Genomics Web includes Tripal ElasticSearch (7). This module allows Tripal sites to index the content they would like to share and provides an interface for cross-site query.

In addition to providing a mechanism for data access across sites, accessible analytical frameworks are required to process large datasets. Tripal Galaxy (https://www.drupal.org/project/tripal_galaxy) provides an API to communicate with any Galaxy server. This web-based platform provides transparent and reproducible research through open source bioinformatic applications and connected workflows (5). Galaxy was first developed to enable access to packages commonly implemented in genomics research and has gained utility across a number of disciplines. The Tripal Galaxy API enables administrators to develop workflows custom to their data and provides guided parameterization as well as management of the required high performance computing resources available locally or remotely. The API does this through the use of the blend4php (https://github.com/galaxyproject/blend4php) library, a wrapper for the Galaxy API written specifically for PHP-based applications such as Drupal.

While Tripal Exchange provides a mechanism for cross-site query and Tripal Galaxy provides an API to an established analytical platform, there remains a need to bring datasets that are several gigabytes to several terabytes in size across the network. This transfer remains one of the more significant bottlenecks for big data manipulation. Tripal Transfer (https://github.com/feltus/BDSS) focuses on optimizing the transfer of large datasets from primary data sources, such as GenBank, as well as other Tripal sites through identification of the optimal path and reliance on hubs with direct connections to Internet2. Efficient data transfer allows the products of cross-site queries to localize at the appropriate Galaxy instance for analysis.

#### Back-end Database and the web interface of TreeGenes

**Figure 2:**
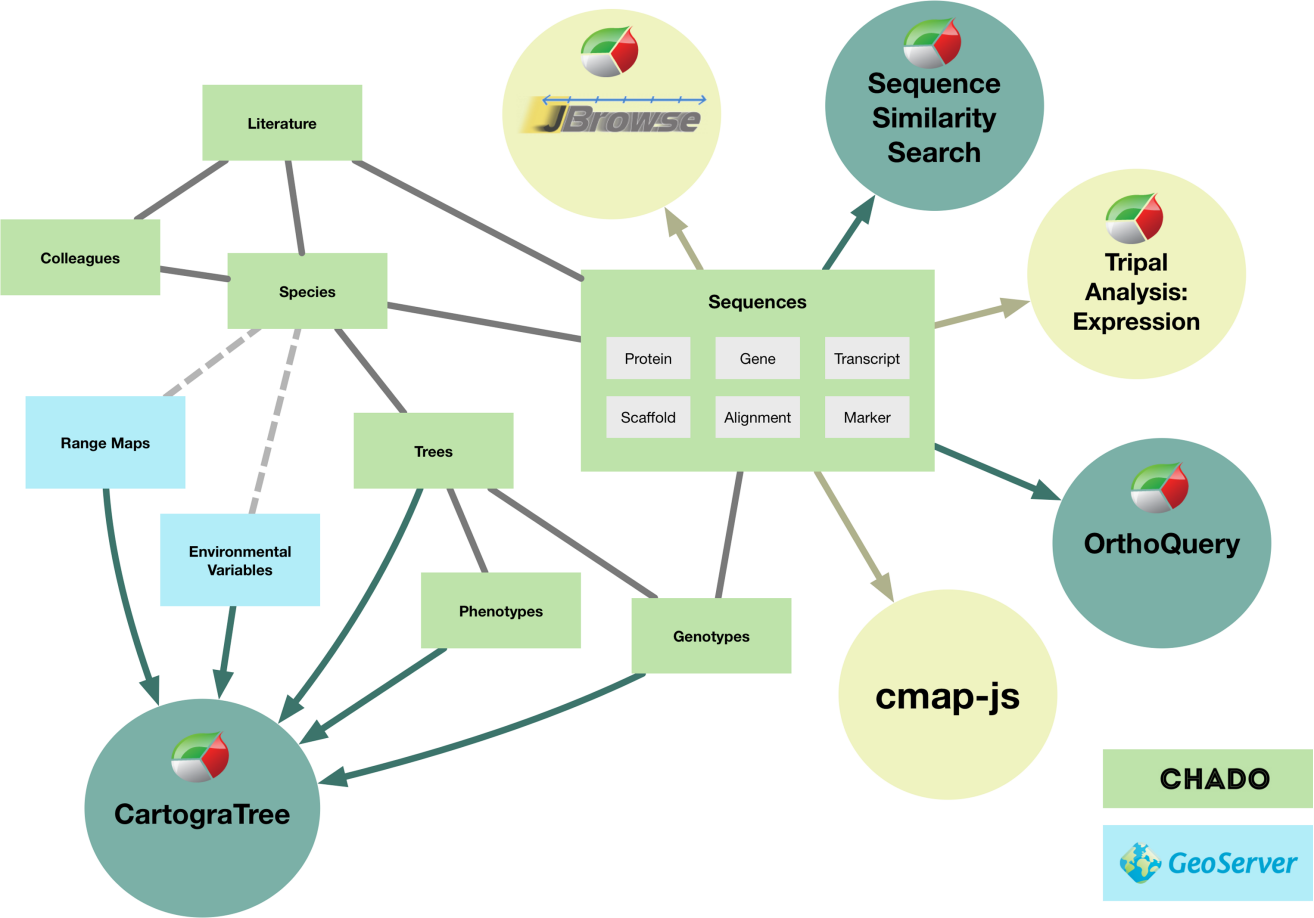
The internal design of Chado and connections to other areas of TreeGenes. Items in green boxes are part of the structure of the Chado relational database. Items in blue are used exclusively for CartograTree, but the module does pull data from multiple parts of the database. Sequence data are displayed or manipulated by a number of tools. The tools developed by TreeGenes are dark green, and the ones implemented by other groups are in light green.

TreeGenes stores data within the Chado schema, organized around three primary content types: colleague, publication and species (18) (Figure 2). The colleague module represents 1093 researchers across 873 institutions worldwide. These colleagues have profiles that define their species of interest, publications, as well as contact details. Currently, a total of 24 847 publications are housed in TreeGenes that are sourced bi-weekly from Web of Science (https://clarivate.com/products/web-of-science/) and manually imported from Dryad (https://datadryad.org/). Colleague information can be linked to these publications and the recent integration of ORCID (https://orcid.org) credentials assists with this authentication. Species records are associated with both colleagues and literature. NCBI’s Taxonomy database serves as the basis for locating information associated with the 1791 species in TreeGenes (8). Addition of a new species into TreeGenes is based upon availability of at least one piece of genetic data from the taxonomic orders of interest. Associated genetic, phenotypic and environmental data are imported from user submissions, as well as primary and secondary data banks based upon the most recent update of literature and species records.

**Figure 3:**
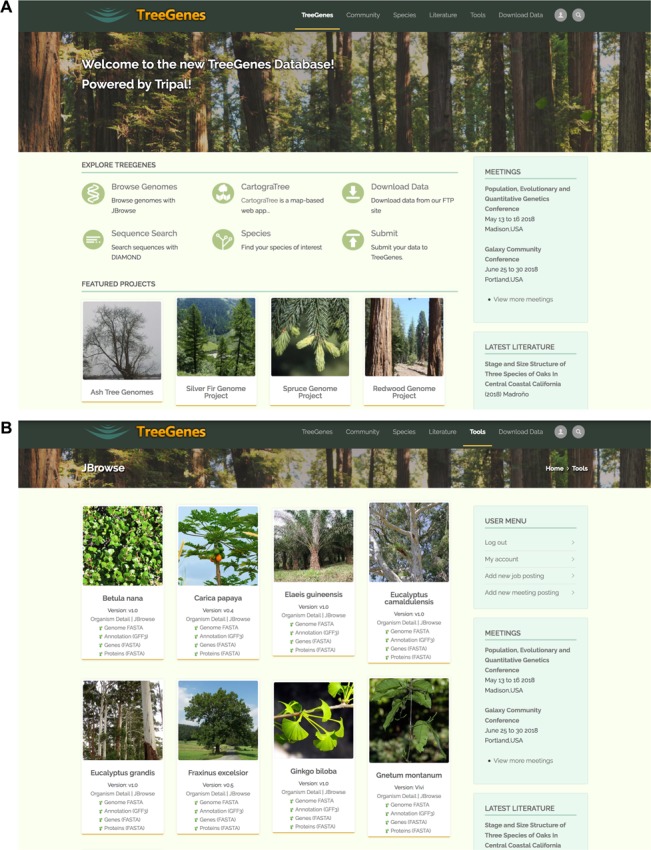
Screen captures of the main TreeGenes webpages. (**A**) The home page of the TreeGenes database has links to various tools such as JBrowse and CartograTree, as well as links to download and search pages. (**B**) The JBrowse page landing page which features access to the viewer as well as associated flat files and short read alignment indexes.

The TreeGenes repository is accessible through any browser pointed at *treegenesdb.org*. Individual pages are designed and maintained through Drupal, which facilitates web forms, summary pages and administrative pages. The homepage provides access to research community relevant lists, current large-scale research collaborations, as well as access to pages serving the related search forms and tools (Figure 3A and B). Data content can be directly queried through the search interfaces via Drupal forms or the ElasticSearch Tripal module which is presented as a search bar on pages representing indexed data types. Both approaches access the TreeGenes data organized in Chado and optimized for query via materialized views. In addition to web-based queries, flat files of sequence sets, including full genomes and indexes for short read aligners acting on these reference genomes, are available on TreeGene’s FTP. Several Tripal extension modules are installed on the website to provide visualization and analytical features.

#### Open source software architecture of TreeGenes

**Figure 4:**
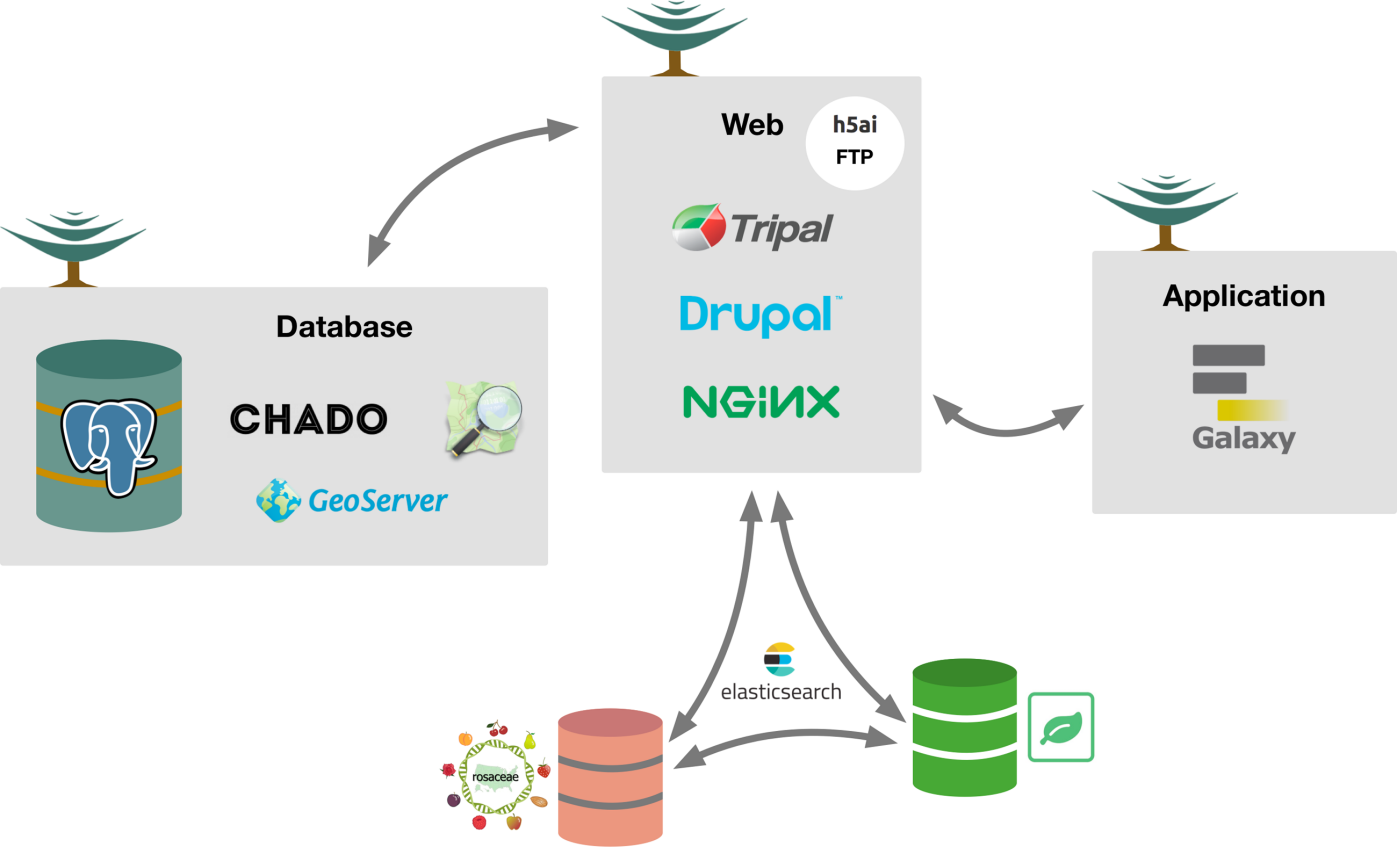
Conceptual overview of the TreeGenes database. The architecture (hardware and software) underlying TreeGenes. There are three distinct servers: database, web and application. The database server, running PostgreSQL, houses Chado and GeoServer. This connects to the web server which houses Tripal, Drupal and the web server software, NGINX, as well as FTP through h5ai. ElasticSearch is installed as an extension module to provide access to indexed content in the database and search indexed content in partner databases (Genome Database for Rosaceae and Hardwood Genomics Web). The web server connects to the app server which hosts Galaxy and gives TreeGenes users access to computational resources.

TreeGenes is distributed across independent and replicated web, database and application servers to promote a development to production model (Figure 4). All hardware is running CentOS 7 Linux. The web servers host: Tripal v3, Drupal v7, NGINX v1.10.2, PHP v7.0.25, CMAP (https://github.com/LegumeFederation/cmap-js), H5AI (https://larsjung.de/h5ai/) and JBrowse (16). The database servers host PostgreSQL v9.5 with the Chado schema and GeoServer v2.11.2. The application server hosts a Galaxy instance v17.09, as well as independent applications responsible for batch processing of large genomic datasets.

### Data sources for TreeGenes

#### Genetics and genomics

NCBI’s GenBank serves as the primary repository for raw sequence data (SRA), reference genomes (WGS and Genome), assembled transcriptomes (TSA), expressed sequence tags (ESTs), complementary DNA (cDNA) and expression studies (GEO). TreeGenes currently hosts 2142 BioProjects, 19 genomes, 2 303 178 TSA records, 588 695 EST records, 188 982 cDNA records, 15 109 808 dbSNP records and 466 GEO studies from GenBank. Aside from GenBank, reference genomes may also be curated from JGI’s Phytozome (9) or Ensembl (10). An additional 14 genomes hosted by TreeGenes are sourced from Phytozome or Ensembl.

While many forest tree species lack reference genome assemblies, approximately 186 species are represented by studies focused on the coding regions of the genome. Community projects, such as 1000 Plants (1KP), provide an important source of information for 87 of the TreeGenes hosted species. From this project and the combined GenBank resources (EST, cDNA, TSA), TreeGenes curates a non-redundant set of UniGenes for 40 species that serve as a comprehensive resource for species specific gene sets.

TreeGenes hosts genotypic data, in the form of SSRs and SNPs for 69 species. This information was previously imported from NCBI’s dbSNP but is currently the product of direct submissions to the database. Population level studies in the form of genotyping assays, exome capture and GBS are the primary sources of variant data.

**Table 1:** Environmental data available in TreeGenes. For each data set, the provider, the name of the data set, its source and a short description are shown, along with the format in which it is stored, and how it can be accessed in TreeGenes

Provider	Environmental layer	Description	Storage	Access
WorldClim v2 (15, 33–39)	Minimum temperature^1^	Average monthly climate data for 1970–2000	GeoTiff files	Raster layers in CartograTree
	Maximum temperature^2^			
	Average temperature^3^			
	Precipitation^4^			
	Solar radiation^5^			
	Wind speed^6^			
	Water vapor pressure^7^			
Conservation Biology Institute (30, 40)	Harmonized World Soil Database^8^	Composition of 15,773 soil mapping units, and standardized soil parameters for top- and subsoil.	PostGIS table	Vector layer in CartograTree
US Forest Service (19, 41)	Species range maps^9^	Distribution maps of 135 tree species	Shape files	Vector layers in CartograTree
US Geological Survey (43)	Tree cover^10^	Estimates for tree cover and bare ground	GeoTiff files	Raster layer in CartograTree
Global Aridity and PET Database (31, 32, 43)	Annual global aridity^11^	Data related to evapotranspiration processes and rainfall deficit for potential vegetative growth	GeoTiff file	Raster layer in CartograTree
NASA (44)	Canopy height^12^	The height of the world’s forests	GeoTiff file	Raster layer in CartograTree
Intact Forest Landscapes (45)	Intact Forest Landscapes^13^	The extent of the intact forest landscapes	Shape files	Vector layers in CartograTree

^1^http://biogeo.ucdavis.edu/data/worldclim/v2.0/tif/base/wc2.0_30s_tmin.zip

^2^http://biogeo.ucdavis.edu/data/worldclim/v2.0/tif/base/wc2.0_30s_tmax.zip

^3^http://biogeo.ucdavis.edu/data/worldclim/v2.0/tif/base/wc2.0_30s_tavg.zip

^4^http://biogeo.ucdavis.edu/data/worldclim/v2.0/tif/base/wc2.0_30s_prec.zip

^5^http://biogeo.ucdavis.edu/data/worldclim/v2.0/tif/base/wc2.0_30s_srad.zip

^6^http://biogeo.ucdavis.edu/data/worldclim/v2.0/tif/base/wc2.0_30s_wind.zip

^7^http://biogeo.ucdavis.edu/data/worldclim/v2.0/tif/base/wc2.0_30s_vapr.zip

^8^http://www.arcgis.com/home/item.html?id=1d16ed2a0aa24ab39e5ee6c491965883

^9^https://www.fs.fed.us/nrs/atlas/littlefia/

^10^https://landcover.usgs.gov/glc/

^11^http://www.cgiar-csi.org/data/global-aridity-and-pet-database

^12^https://landscape.jpl.nasa.gov/

^13^http://www.intactforests.org/data.ifl.html

#### Phenotypes and phenomics

Phenotypic terms and their associated measures are primarily collected and curated from direct submissions by authors to TreeGenes. Phenotypes are accepted for trees that have exact as well as approximate georeferenced locations. Additional phenotypic metrics are collected from three independent sources: TRY-DB, Dryad and TreeSnap (https://treesnap.org/). The international web-based repository TRY-DB stores plant trait terms available from over 400 researcher-contributed databases (11). The TreeGenes database has imported 6049 unique terms from 693 tree species from TRY-DB to date. As a consumer and an accession provider of data associated with peer reviewed journals, Dryad has become an important source of phenotypic (as well as associated genotypic) data in TreeGenes representing a total of 33 studies. Dryad is widely adopted by ecology and evolutionary biology journals and a subset of submissions can be curated for phenotypic metrics for georeferenced tree species. The variable file format options in Dryad require a manual curation process before data can be imported to the database. In addition to curated phenotypic data, the citizen science and land manager focused mobile application, TreeSnap, provides a venue for identifying and reporting phenotypic measures for specific forest tree species. TreeGenes routinely imports validated and georeferenced forest trees accessions from TreeSnap, and has collected data on 255 trees (68 species) to date.

#### Environmental data

TreeGenes stores georeferenced environmental data, which is crucial for interrogating genotypic data in diverse forest tree populations (Table 1). TreeGenes curates and hosts global environmental data from public repositories and local data uploaded by authors prior to publication. These data can be accessed from the custom extension module, CartograTree (12).

#### Community submissions and standards

**Figure 5:**
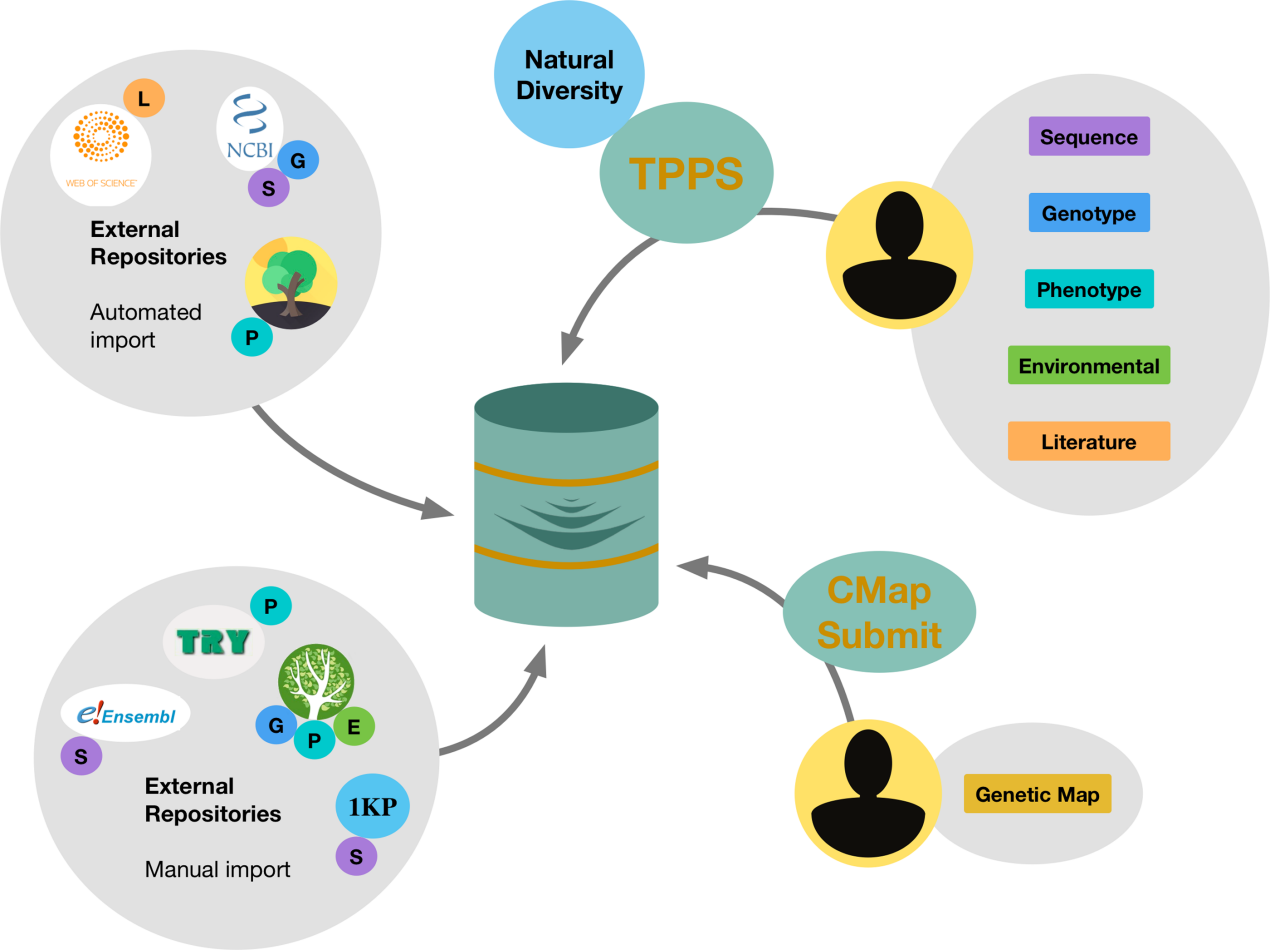
Submission and data acquisition avenues for the TreeGenes database. Clockwise from top left: TreeGenes automatically imports literature data from Web of Science, sequence and genotypic information from GenBank and georeferenced phenotypic data from TreeSnap; users can submit genotypic, phenotypic, environmental and literature data through the TPPS platform; users can also submit genetic maps through the CMap pipeline; TreeGenes managers manually import sequence data from Ensembl and 1KP, phenotypic data from TRY-DB and georeferenced genotypic and phenotypic data from Dryad.

In this past 5 years alone, over 670 studies have been published on association genetics and/or landscape genomics of forest trees. Very little of this data is formally collected as georeferenced accessions with full integration of genotype and phenotype. TreeGenes has developed the Tripal Plant Pop-Gen Submit pipeline (TPPS) to specifically capture data and metadata describing genotype, phenotype and environmental studies associated with landscape genomics or association genetics investigations (Figure 5). The workflow relies on a series of questions to properly describe the experimental design, including location, replication and treatments. These questions also guide the system on the types of raw and intermediate data to request. Raw sequence data and reference genomes are sent to the primary repositories and linked back to TreeGenes via NCBI/EBI accession numbers. Intermediate deliverables, such as assemblies and genotypes, are accepted within the TPPS workflow. TPPS is able to accommodate a wide range of designs common to forest genetics studies: landscape sampling, breeding plots, common gardens and growth chamber experiments.

TPPS enforces minimal reporting standards and associated biological ontologies to provide reusable data. The module employs standards established as the Minimal Information About a Plant Phenotyping Experiment (MIAPPE) to guide the collection of phenotypic data as well as the overall experimental design (17). The MIAPPE standards were developed from the objectives of transPLANT, European Plant Phenotyping Network and ELIXIR-EXCELERATE projects with the goal of developing reporting requirements to describe plant phenotyping experiments. MIAPPE integrates its minimal reporting standards with existing ontological frameworks. TreeGenes has implemented five of these: Plant Ontology (PO) (14), Chemical Entities of Biological Interest (ChEBI) (33), Trait Ontology (TO) (34), Crop Ontology (CO) (13), Phenotype And Trait Ontology (PATO) (https://github.com/pato-ontology/pato) and a custom TreeGenes ontology that serves to hold traits in transition to established ontologies (Table 2). Plant structure, development and trait terms are integrated with PATO and supporting ontologies via the Planteome project which enables comparative biology across the omics. In addition to the traits and their associated plant structures, genotypic values are collected through TPPS. Currently, TPPS can accommodate both SNPs and microsatellites (SSRs). This marker data is collected in the context of the sequencing design, which may include genotyping assays, genotype-by-sequencing (GBS) approaches, transcriptomic sequencing and whole genome resequencing. Community standards, such as the Variant Common Format, are preferred. However, alternatives consistent with minimal reporting are accepted and will be converted and stored for re-distribution in standard file formats. For all submissions with genotypic values, TPPS requires the user to reference a genome and version, or provide an intermediate assembly (transcriptomic or genomic) from which the SNP calls were derived. In a final, optional step, environmental data for the georeferenced trees can be loaded directly from the layers used or as independent measurements conducted by the investigators.

**Table 2:** Ontology frameworks and statistics for TreeGenes

Ontology	Unique Terms	Total Measures
PO	23	724 225
TO	20	227 338
CO	19	173 066
ChEBI	126	42 412
PATO	21	642 326
TreeGenes Ontology (TGDR)	5	1745

Following acceptance and validation of the data in the TPPS module, an accession number is supplied that provides a long-term reference to the entire dataset. TreeGenes works closely with journals focused on tree genetics to encourage researchers to submit these data at the time of publication. To date, TreeGenes has collected a total of 71 studies representing 56 species. The combination of TPPS submissions as well as imported information from TRY-DB, Dryad and TreeSNAP represents 36 730 176 genotypes and 935 596 phenotype records. Accepted studies are available in TreeGenes under the ‘Tripal Plant Pop-Gen Submissions’ page, where users can download the associated flat files, organized by content type.

### Tools for query, submission, curation, analysis and visualization

One of the main features of Tripal is its modular design, which allows developers to easily create and publish custom modules for others to use and install extensions developed by other members in the community. TreeGenes takes advantage of this feature and provides its users a number of Tripal extension modules developed by either the community or in-house, which enables the researchers to stay entirely within the site executing an analysis. Custom modules allow users to submit, query, analyze and visualize data all within TreeGenes.

To query and analyze data in TreeGenes, users have several options, enabled by the ElasticSearch module, Tripal Sequence Similarity Search (TSeq) module and the upcoming OrthoQuery module. With ElasticSearch, users can perform text searches in TreeGenes, similar to a standard web browser. Any table exposed by TreeGenes can be searched using this module. For example, searching for ‘drought’ returns 179 publications that contain this term in their title, abstract or keywords. The results are shown over several pages, with part of the abstract of each publication listed, the term highlighted in bold font and a link to the publication page in TreeGenes. Moreover, ElasticSearch enables fast and scalable searching across the Tripal sites it is installed on. Currently, ElasticSearch is connected to the Hardwood Genomics Project database, allowing users to query available gene indexes, species, phenotypes, sequence records and more. Once additional Tripal databases are connected, such as the Genome Database for Rosaceae and Citrus Genome Database, users and researchers will be able to pull in an expansive set of data and search through multiple sites and databases at once (4).

Sequence search functionality is provided by TSeq in TreeGenes. TSeq provides access to the speed increase available through Diamond (35) for BLASTP/BLASTX style searches as well as traditional NCBI BLAST (36) for BLASTN. Both applications are integrated into a single interface that provides file upload or copy/paste sequence support for the query and access to formatted databases for NCBI BLAST or Diamond. The target databases can be customized for the categories of whole genome, gene, protein and transcriptome/UniGene. TSeq leverages Tripal Remote Job, another Tripal Extension module from the community, to run jobs on external machines with high computational capacity. The output is in the familiar BLAST XML format and can be viewed in a table or downloaded for the user’s convenience.

OrthoQuery is being developed for comprehensive comparative genomic analysis using orthogroups. Comparisons across orthogroups can help characterize selection pressure, evolutionary rate of specific gene families, novel gene families and identify whole or partial genome duplication events even in the absence of reference genome. Tools like OrthoFinder (20) can execute such comparisons and investigate relationships among sets of protein sequences. These tools are however limited, as they do not allow users to select and filter datasets, or provide a robust visualization and query interface. OrthoQuery is a Tripal module that provides a semi-automated analytical pipeline and visualization platform. It identifies orthologous genes from user-selected UniGenes represented in the database, executes OrthoFinder optimized with Diamond and utilizes the Tripal framework with Galaxy integration to support execution of multiple workflows. Visualization features are enabled by building a customized D3 script (https://d3js.org/) that allows the user to interactively interrogate specific gene families based on phylogenetic relationships and query the specified orthogroups.

Visualization of genomic data residing in TreeGenes is supported by two community-developed modules, JBrowse and Natural Diversity Genotypes (https://www.drupal.org/project/nd_genotypes). With JBrowse, users can visualize, consult and analyze multiple genomes that are held in the TreeGenes database (Figure 3B). JBrowse is an online genome browser built in HTML5 and JavaScript that provides a low latency tool for the visualization of genomes and genome annotation. The Natural Diversity Genotypes module adds a specialized interface for displaying information from the central tables in a Chado database schema. A search view, genotype view and sequence view create a more usable experience for users looking to summarize this information. This module also provides genotype search functionality such that users can select individuals and variants of interest, followed by the option of downloading the desired dataset as a tab-delimited file.

CartograTree is a visualization and analysis module for meta-analysis of genotype, phenotype and environmental data of georeferenced tree accessions derived from association genetics and landscape genomics studies. It is challenging for researchers to identify and combine the resources necessary for meta-analysis of landscape genomics and association mapping studies. Hurdles include different projections of global information system (GIS) data, improper formatting of source files or incomplete genotype and phenotype metrics. CartograTree leverages the TreeGenes database to present genetic and phenotypic data in a simple, visual interface for researchers, and create visualizations based on results. Trees can be queried by species or study affiliation directly through the tool or loaded into CartograTree via links provided from species detail pages and the TPPS landing page. TPPS submitted studies provide direct access to the genotypic and phenotypic values. CartograTree also provides an interface for properly formatted environmental data, allowing researchers to focus on designing the workflows to integrate biotic and abiotic data. These analyses can be supported within CartograTree since it integrates with the Galaxy platform that is home to a variety of bioinformatic tools and workflows.

## Conclusions and future directions

The TreeGenes database has served the forest genetics community since the early 1990s. It was implemented first as Dendrome, a repository to hold information related to genetic linkage maps and the researchers who generated them. Over the years, the database embraced a relational model to store data and expanded to include data submitted not only by its own users, but also information curated by numerous primary and secondary sources. Recent development has focused on providing an integrated platform that contains genetic information, as well as the associated phenotype and environmental information. In the era of high-throughput sequencing, TreeGenes adopted more efficient models for data storage and developed custom interfaces that were focused on retrieving larger datasets or executing sequence similarity searches for numerous sequences. The recent transition to the Tripal framework was fueled by the desire to develop in a more flexible and sustainable platform. Tripal provides a framework that can adapt to new data types on the back end and provide an interface that can be quickly customized on the front end. Migration to Tripal also provided membership to a community of over 100 databases, many of which are supporting plant-focused research. The Tripal Gateway framework, available in version 3, has provided functionality to support cross-site query, efficient data transfer and access to analytical pipelines, such as Galaxy. The TreeGenes database is unique in its wide species representation and minimal reference sequence resources. For its community, TreeGenes has pursued comparative genomics and population genomics through the development of Tripal extension modules, such as TPPS, TSeq, OrthoQuery and CartograTree.

Future development in TreeGenes is focused on advanced metadata integration, analytical pipelines that can work with user provided data and further integration with partner databases to achieve improved visualization for comparative genomics. The field of genomics will continue to evolve with sequencing technologies that can generate more reference material and assess even larger populations. The field of phenomics, especially for forest biology, is moving toward remote sensing technologies to provide high-throughput phenotypic for forest health and production. If developers combine these in an integrated environment with appropriate metadata and analytical capacity, the research impact will be tremendous.

## Notes

**Database URL**: https://treegenesdb.org/Drupal

## References

[1] Aitken S.N. and Bemmels J.B. (2016) Time to get moving: assisted gene flow of forest trees. Evol. Appl., 9, 271–290 10.1111/eva.12293. 27087852 PMC4780373

[2] Sanderson L.-A., Ficklin S.P., Cheng C.-H. et al. (2013) Tripal v1.1: a standards-based toolkit for construction of online genetic and genomic databases. Database, 2013, bat075–bat075. 24163125 10.1093/database/bat075 PMC3808541

[3] Mungall C.J. and Emmert D.B. (2007) A Chado case study: an ontology-based modular schema for representing genome-associated biological information. Bioinformatics, 23, i337–i346. 17646315 10.1093/bioinformatics/btm189

[4] Jung S., Ficklin S.P., Lee T. et al. (2014) The genome database for Rosaceae (GDR): year 10 update. Nucleic Acids Res., 42, D1237–D1244. 24225320 10.1093/nar/gkt1012 PMC3965003

[5] Afgan E., Baker D., Beek M. van den et al. (2016) The Galaxy platform for accessible, reproducible and collaborative biomedical analyses: 2016 update. Nucleic Acids Res., 44, W3–W10. 27137889 10.1093/nar/gkw343 PMC4987906

[6] Kremer A., Abbott A.G., Carlson J.E. et al. (2012) Genomics of Fagaceae. *Tree Genet.* Genomes, 8, 583–610.

[7] Chen M., Henry N., Almsaeed A. et al. (2017) New extension software modules to enhance searching and display of transcriptome data in Tripal databases. Database, 2017. 10.1093/database/bax052 PMC5532966 29220446

[8] Benson D.A., Cavanaugh M., Clark K. et al. (2018) GenBank. Nucleic Acids Res., 46, D41–D47. 29140468 10.1093/nar/gkx1094 PMC5753231

[9] Goodstein D.M., Shu S., Howson R. et al. (2012) Phytozome: a comparative platform for green plant genomics. Nucleic Acids Res., 40, D1178–D1186. 22110026 10.1093/nar/gkr944 PMC3245001

[10] Aken B.L., Achuthan P., Akanni W. et al. (2017) Ensembl 2017. Nucleic Acids Res., 45, D635–D642. 27899575 10.1093/nar/gkw1104 PMC5210575

[11] Kattge J., Díaz S., Lavorel S. et al. (2011) TRY - a global database of plant traits. Glob. Chang. Biol., 17, 2905–2935.

[12] Herndon N., Grau E.S., Batra I. et al. CartograTree: enabling landscape genomics for forest trees In: Marchesini I. & Pierleoni A. (eds). *Proceedings of the 4th Open Source Geospatial Research and Education Symposium (OGRS2016)*, Perugia, 12–14 October 2016. 10.30437/ogrs2016_paper_34

[13] Shrestha R., Matteis L., Skofic M. et al. (2012) Bridging the phenotypic and genetic data useful for integrated breeding through a data annotation using the Crop Ontology developed by the crop communities of practice. Front. Physiol., 3, 326. 22934074 10.3389/fphys.2012.00326 PMC3429094

[14] Cooper L., Meier A., Laporte M.-A. et al. (2018) The Planteome database: an integrated resource for reference ontologies, plant genomics and phenomics. Nucleic Acids Res., 46, D1168–D1180. 29186578 10.1093/nar/gkx1152 PMC5753347

[15] Fick S.E. and Hijmans R.J. (2017) WorldClim 2: new 1-km spatial resolution climate surfaces for global land areas. Int. J. Climatol., 37, 4302–4315.

[16] Buels R., Yao E., Diesh C.M. et al. (2016) JBrowse: a dynamic web platform for genome visualization and analysis. Genome Biol., 17, 66. 27072794 10.1186/s13059-016-0924-1 PMC4830012

[17] Ćwiek-Kupczyńska H., Altmann T., Arend D. et al. (2016) Measures for interoperability of phenotypic data: minimum information requirements and formatting. Plant Methods, 12, 44. 27843484 10.1186/s13007-016-0144-4 PMC5103589

[18] Mungall C.J., Emmert D.B., Gelbart W.M. et al. (2007) A Chado case study: an ontology-based modular schema for representing genome-associated biological information *Bioinformatics*; Oxford University Press, 23, i337–i346. 10.1093/bioinformatics/btm189 17646315

[19] Prasad A.M. and Iverson L.R. (2003). Little's range and FIA importance value database for 135 eastern US tree species. Northeastern Research Station, USDA Forest Service, Delaware, Ohio http://www.fs.fed.us/ne/delaware/4153/global/littlefia/index.html.

[20] Emms D.M. and Kelly S. (2015) OrthoFinder: solving fundamental biases in whole genome comparisons dramatically improves orthogroup inference accuracy. Genome Biol., 16, 157. 26243257 10.1186/s13059-015-0721-2 PMC4531804

[21] Li L., Stoeckert C.J. and Roos D.S. (2003) OrthoMCL: identification of ortholog groups for eukaryotic genomes. Genome Res., 13, 2178–2189. 12952885 10.1101/gr.1224503 PMC403725

[22] Dhanapal A.P. and Govindaraj M. (2015) Unlimited thirst for genome sequencing, data interpretation, and database usage in genomic era: the road towards fast-track crop plant improvement. Genet. Res. Int., 2015, 1–15. 10.1155/2015/684321 PMC4383144 25874133

[23] Ludovisi R., Tauro F., Salvati R. et al. (2017) UAV-based thermal imaging for high-throughput field phenotyping of black poplar response to drought. Front. Plant Sci., 8, 1681. 29021803 10.3389/fpls.2017.01681 PMC5623950

[24] Skinner M.E., Uzilov,A.V, Stein L.D. et al. (2009) JBrowse: a next-generation genome browser. Genome Res*.*, 19, 1630–1638. 19570905 10.1101/gr.094607.109 PMC2752129

[25] Wheeler N.C., Steiner K.C., Schlarbaum S.E. et al. (2015) The evolution of forest genetics and tree improvement research in the United States. J. For., 113, 500–510.

[26] Stein L.D. (2010) The case for cloud computing in genome informatics. Genome Biol, 11, 207. 20441614 10.1186/gb-2010-11-5-207 PMC2898083

[27] Wegrzyn J.L., Lee J.M., Tearse B.R. et al. (2008) TreeGenes: a forest tree genome database. *Int. J. Plant Genomics*, 2008, 412875. 18725987 10.1155/2008/412875 PMC2517852

[28] Wegrzyn J.L., Main D., Figueroa B. et al. (2012) Uniform standards for genome databases in forest and fruit trees. Tree Genet. Genomes, 8, 549–557.

[29] Neale D.B. and Kremer A. (2011) Forest tree genomics: growing resources and applications. Nat. Rev. Genet., 12, 111–122. 21245829 10.1038/nrg2931

[30] FAO/IIASA/ISRIC/ISS-CAS/JRC, 2009. Harmonized World Soil Database (version 1.1). FAO, Rome, Italy and IIASA, Laxenburg, Austria.

[31] Zomer R.J., Trabucco A., Bossio D.A. et al. (2008) Climate change mitigation: a spatial analysis of global land suitability for clean development mechanism afforestation and reforestation. Agric. Ecosystems Environ., 126, 67–80.

[32] Zomer R.J, Bossio D.A, Trabucco A. et al. (2007). Trees and water: smallholder agroforestry on irrigated lands in Northern India. Colombo, Sri Lanka: International Water Management Institute. IWMI Research Report 122. p. 45.

[33] Hastings J., Owen G., Dekker A. et al. (2016) ChEBI in 2016: improved services and an expanding collection of metabolites. Nucleic Acids Res., 44, D1214–D1219. 26467479 10.1093/nar/gkv1031 PMC4702775

[34] Cooper L., Meier A., Laporte M.A. et al. (2018) The Planteome database: an integrated resource for reference ontologies, plant genomics and phenomics. Nucleic Acids Res., 46, D1168–D1180. 29186578 10.1093/nar/gkx1152 PMC5753347

[35] Buchfink B., Xie C. and Huson D.H. (2014) Fast and sensitive protein alignment using DIAMOND. Nat. Methods, 12, 59–60. 25402007 10.1038/nmeth.3176

[36] Boratyn G.M., Schäffer A.A., Agarwala R. et al. (2012) Domain enhanced lookup time accelerated BLAST. *Biol.* Direct, 7, 12. 22510480 10.1186/1745-6150-7-12 PMC3438057

